# Assessment of pazopanib-related hypertension, cardiac dysfunction and identification of clinical risk factors for their development

**DOI:** 10.1186/s40959-017-0024-8

**Published:** 2017-06-30

**Authors:** Daniel Pinkhas, Thai Ho, Sakima Smith

**Affiliations:** 1 Department of Internal Medicine, The Ohio State University Wexner Medical Center, 395 West 12th Avenue, Third Floor, Columbus, OH 43210 USA; 2 Division of Hematology and Medical Oncology, Mayo Clinic, Scottsdale, AZ USA; 3 Division of Cardiology, Advanced Heart Failure, Cardiac Transplantation, and Mechanical Circulatory Support, Columbus, OH USA

**Keywords:** Cardiotoxicity, Pazopanib, Vegf, Hypertension, Angiogenesis inhibitors, Small molecule tyrosine kinase inhibitors, Vascular toxicity, Cardio-oncology

## Abstract

**Background:**

Antineoplastic therapy with the tyrosine kinase inhibitor pazopanib in patients with advanced/metastatic renal cell carcinoma (mRCC) has been associated with hypertension (HTN), cardiomyopathy, and cardiac dysrhythmias. We therefore assessed the cardiovascular (CV) risk with pazopanib in a clinical setting.

**Methods:**

Medical records of 35 antineoplastic-naïve mRCC patients newly started on pazopanib were retrospectively reviewed at a single academic medical center. Assessment of the hypertensive response and adverse cardiac events associated with pazopanib was the primary objective. Outcomes were defined using the National Cancer Institute’s Common Terminology Criteria for Adverse Events v4.0. Potential clinical risk factors were investigated with univariate and multivariable logistic regression.

**Results:**

Pazopanib-induced HTN was observed in 57% of patients. Median maximal systolic blood pressure (SBP) during pazopanib treatment was 167.5 mmHg with median time to event of 24.5 days. New-onset HTN occurred in 6/14 (43%) patients. Baseline SBP > 130 mmHg (odds ratio [OR]: 5.32; 95% confidence interval [CI]: 0.94-29.99; *p* = 0.058) and ACEi/ARB use (OR: 4.88; 95% CI: 1.05 22.84; *p* = 0.044) were risk factors for pazopanib-induced HTN. When HTN was excluded, 34% of patients developed a CV adverse event. Age ≥ 60 years (OR: 8.72; 95% CI: 0.74-513.26; *p* = 0.105) trended towards being a predictor for a non-HTN CV adverse event.

**Conclusions:**

Our findings suggest that pazopanib has a broad CV toxicity profile in treatment-naïve mRCC patients headlined by a rapid and striking hypertensive response. More intensive BP control prior to starting pazopanib and standardization of CV surveillance particularly in older patients may optimize oncologic care while minimizing CV risk.

## Background

Vascular endothelial growth factor (VEGF) signaling pathway inhibitors (VSPI) have known efficacy in multiple malignancies by inhibiting tumor angiogenesis, but are increasingly being recognized as cardiotoxins. Small-molecule targeted VEGF receptor (VEGFR) tyrosine kinase inhibitors (TKI) have significantly improved outcomes in advanced/metastatic renal cell carcinoma (mRCC) as evidenced by 6 and 14 month increases in median progression free survival and overall survival, respectively, with sunitinib compared to earlier first line agents for mRCC [1, 2]. Pazopanib is a newer oral angiogenesis inhibitor targeting VEGFR-1, −2, and −3, platelet-derived growth factor receptor (PDGFR)-α and -β, and c-KIT and has been a first-line agent for mRCC since 2009 [3–5]. Potential expansion of pazopanib’s use in various pediatric and adult malignancies is currently being investigated [6]. Pazopanib has similar efficacy compared to sunitinib, a similar multi-targeted VSPI that preceded it and whose cardiotoxic effects are described most often within this class [7, 8]. Within its class, pazopanib’s favorable overall side effect profile and cost-effectiveness have made it an appealing option for physicians and patients [9–13]. Consideration of these factors suggests that an increasingly higher number of patients with mRCC will be treated with pazopanib.

Like other anti-neoplastic VSPIs, pazopanib has been associated with a cardiovascular (CV) toxicity profile that includes arterial hypertension (HTN), ischemic and thrombotic events, cardiomyopathy, and cardiac dysrhythmias [14–18]. Among these, HTN is by far the most common with a reported 35.9% incidence among pazopanib-treated patients [19]. In 362 pazopanib-treated patients, a 1% incidence of symptomatic heart failure (HF) and 9% incidence of an absolute left ventricular (LV) ejection fraction (LVEF) decline of 15% or greater was observed [20]. Higher rates were described in a meta-analysis that included 3 trials (*n* = 314) and found a HF incidence rate of 6.1% [21]. Pazopanib-related conduction disturbances reported in phase 3 clinical trials included QT prolongation >500 milliseconds (ms) and Torsades de pointes at incidences of <2% and <1%, respectively [22]. In addition, there are case reports describing pazopanib-related apical ballooning syndrome and rapidly progressive fulminant heart failure [23, 24].

Roughly 63,000 patients in the United States are diagnosed with renal cancer annually [25]. Given that the median age of diagnosis of RCC is 64 years, many of them have an increased risk or may already have preexisting CV disease prior to initiating targeted VSPI treatment such as pazopanib [26]. Development of clinically significant HTN can result in morbidity and pazopanib dose reduction or cessation, thus limiting the overall efficacy of cancer treatment. Our objective was to characterize the extent of CV toxicity associated with pazopanib and the risk factors for its development in an antineoplastic-treatment naïve, real-world mRCC patient population to capture pazopanib’s unique CV effects.

## Methods

### Study participants

Cases were selected from 462 consecutive male and female patients, age 18 years or greater, with a diagnosis of mRCC. International Classification of Diseases – 9 and 10 (ICD-9/10) diagnosis codes were used to identify cases. All patients had been treated with pazopanib within the Ohio State University Wexner Medical Center (OSUWMC) health system at some point during the period 12/01/2009 to 08/01/2016 and had at least two follow-up visits with an OSUWMC clinician during pazopanib therapy. Cases were excluded if baseline blood pressure (BP) was missing, pazopanib therapy was stopped fewer than 7 days after initiation, or if the patient underwent treatment with any other systemic antineoplastic agent prior to pazopanib exposure. This excluded 427 patients and the 35 remaining patients comprised the final cohort for this study. All 35 patients were followed-up until either death occurred or until their last encounter with an OSUWMC clinician. Follow up was completed in August 2016. The study was approved by the Ohio State University (OSU) Cancer Institutional Review Board.

Baseline patient characteristics were captured using OSUWMC electronic medical records (EMR). This included past medical history elements and medication lists provided at each oncologic-related visit. Study entry date was set as the time of first pazopanib order placed in the EMR. Baseline characteristics included age at pazopanib start date, Eastern Cooperative Oncology Group (ECOG) performance status, tumor histology, starting pazopanib dose, preexisting comorbidities, medications, smoking status, and body mass index (BMI). Cardiovascular comorbidities of interest were HTN, diabetes, dyslipidemia, renal insufficiency defined as a glomerular filtration rate less than 60 ml/min/1.73 m^2^, coronary artery disease, peripheral arterial disease, congestive heart failure, left ventricular dysfunction, cardiac dysrhythmias, cerebrovascular disease, and thromboembolic disease. We identified use of angiotensin-converting enzyme inhibitors (ACEi), angiotensin-receptor blockers (ARBs), beta-blockers (BBs), calcium-channel blockers (CCBs), diuretics, statins, and metformin at or before study entry date. Medications were followed longitudinally for the entirety of the study period using medication lists available with every oncology office visit within the OSUWMC James Cancer Hospital. Criteria used to assess ongoing pazopanib treatment were a minimum of two related visits in which pazopanib appeared in the medication list. Validation by manual chart review of patient medication lists and oncologic provider documentation in the EMR was performed for each patient to ensure accuracy of pazopanib treatment dates.

### Cardiovascular data review

The primary source for clinical variables was EMR data entered by trained healthcare professionals for clinical purposes within the OSUWMC system. Baseline systolic (S) and diastolic (D) BP was determined using the mean value of measurements obtained at each oncologic office visit in the preceding 90 days of pazopanib start date for each patient. SBP and DBP after pazopanib initiation was determined using the mean value of measurements obtained at each subsequent oncology-related office visit which at minimum included two visits (one at two weeks post-pazopanib initiation and one at four weeks post-pazopanib initiation). Baseline left ventricular ejection fraction (LVEF) was available for 25 out of the total 35 patients in this study. Echocardiography data was obtained from final reports that were only available after an official interpretation was entered by an expert cardiologist. Baseline LVEF determination was with conventional 2-dimensional (2D) echocardiography using the Simpson biplane technique, according to the American Society of Echocardiography guidelines [27]. Baseline electrocardiographs (ECG) were available for review within the EMR in 29 patients. An expert cardiologist had previously confirmed each ECG interpretation. Data extracted were the corrected QT-interval (QTc) derived from Bazett’s formula (QTc = QT/√RR) and the QRS duration. ECGs were manually reviewed and excluded if they had features such as an electronically paced rhythm and/or significant intra-ventricular conduction delay that do not allow for accurate QTc measurement.

### Definition of outcomes

The development of pazopanib-induced HTN was the primary outcome studied. Patients with or without preexisting HTN could meet criteria for the primary outcome. Patients were considered having preexisting HTN if they met any one of the following criteria prior to the date of pazopanib initiation: (a) HTN documented as a diagnosis in the EMR (b) at least one prescribed medication within the antihypertensive class (c) systolic blood pressure (SBP) greater than or equal to 140 mmHg or a diastolic blood pressure (DBP) greater than or equal to 90 mmHg at least two separate clinical encounters. These parameters were chosen in accordance with the National Cancer Institute’s Common Terminology Criteria for Adverse Events (CTCAE v4.0) and Joint National Committee on Prevention, Detection, and Treatment of High Blood Pressure (JNC) 8 [28, 29].

For those without preexisting HTN, pazopanib-induced HTN was defined as the occurrence of any one of the above criteria during pazopanib treatment. For patients with preexisting HTN, pazopanib-induced HTN was defined as any one of the following interventions during pazopanib therapy: (a) addition of a new antihypertensive medication, (b) dose escalation of a baseline antihypertensive medication. Severity of HTN was also assigned based on CTCAE v4.0 definitions, graded 1 to 5 according to severity [29]. CV adverse events (AE) were defined in accordance with the CTCAE v4.0 definitions due its universal acceptance in defining AEs in oncologic clinical trials. CV AEs chosen for inclusion in this study were “Hypertension,” “Heart Failure,” “Electrocardiogram QT corrected interval prolonged,” “Atrial flutter,” and “Peripheral Ischemia”. Table 1 lists all definitions of AEs and severity grades (1 to 5, in ascending severity). Any AE with a grade of 3, 4, or 5 was considered a high-grade event in accordance with the CTCAE.

**Table 1:** Study definitions and severity grades for pazopanib-related cardiovascular adverse events

Hypertension
Grade 1: Pre-hypertension (systolic BP 120–139 mmHg or diastolic BP 80–89 mmHg)
Grade 2: Stage 1 hypertension (systolic BP 140–159 mmHg or diastolic BP 90–99 mmHg); medical intervention indicated; recurrent or persistent (≥24 h); symptomatic increase by >20 mmHg (diastolic) or to >140/90 mmHg if previously within normal limits; monotherapy indicated
Grade 3: Stage 2 hypertension (systolic BP ≥160 mmHg or diastolic BP ≥100 mmHg); medical intervention indicated; more than 1 drug or more intensive therapy than previously used indicated
Grade 4: Life-threatening consequences (e.g., malignant hypertension, transient or permanent neurologic deficit, hypertensive crisis); urgent intervention indicated
Grade 5: Death
Heart Failure
Grade 1: Asymptomatic with laboratory (e.g., BNP) or cardiac imaging abnormalities
Grade 2: Symptoms with mild to moderate exertion
Grade 3: Severe with symptoms at rest or with minimal activity or exertion, intervention indicated
Grade 4: Life-threatening consequences; urgent intervention indicated (e.g., continuous IV therapy or mechanical hemodynamic support)
Grade 5: Death
Electrocardiogram QT corrected interval (QTc) prolonged
Grade 1: QTc 450 – 480 ms
Grade 2: QTc 481 – 500 ms
Grade 3: QTc > = 501 ms on at least two separate ECGs
Grade 4: QTc > = 501 or >60 ms change from baseline and Torsade de pointes or polymorphic ventricular tachycardia or signs/symptoms of serious arrhythmia.
Atrial flutter
Grade 1: Asymptomatic, intervention not indicated
Grade 2: Non-urgent medical intervention indicated
Grade 3: Symptomatic and incompletely controlled medically, or controlled with device (e.g., pacemaker), or ablation
Grade 4: Life-threatening consequences; urgent intervention indicated
Grade 5: Death
Peripheral ischemia
Grade 1: Not defined
Grade 2: Brief (< 24 h) episode of ischemia managed non-surgically and without permanent deficit
Grade 3: Recurring or prolonged (> = 24 h) and/or invasive intervention indicated
Grade 4: Life-threatening consequences; evidence of end organ damage; urgent operative intervention indicated
Grade 5: Death

Adapted from the National Cancer Institute’s Common Terminology Criteria for Adverse Events version 4.0 (CTCAE v4.0) [29]. Per the CTCAE original document, a semi-colon indicates ‘or’ within the description of the grade. High-grade adverse events discussed in the text refer to any event assigned a grade of 3, 4, or 5

*BP* blood pressure, *BNP* brain natriuretic peptide, *IV* intravenous, *QTc* correct QT interval, *ECG* electrocardiogram

### Statistical analysis

Continuous variables are presented as means with standard or as medians with interquartile ranges ([IQR]: 25th-75th percentile.) Categorical variables were compared using Student’s *t* test, Mann-Whitney *U* test, chi-square test, or Fisher’s exact test as appropriate. Univariate logistic regression analysis was performed to estimate odds ratios (ORs) of potential risk factors for the development of pazopanib-induced HTN. Any clinical variables identified in this analysis with *P* < 0.1 were entered into a multivariable logistic regression model to identify independent factors associated with development of pazopanib-induced HTN. In addition to analyzing SBP, age, and BMI as continuous variables, binary variables were established by dichotomizing SBP (above or below 130 mmHg), age (above or below 60 years), and BMI (above or below 30 kg/m^2^) for logistic regression analysis. ORs with 95% confidence intervals (CIs) were generated. Overall survival outcomes were assessed using the log-rank test and Kaplan Meier survival estimates. For all comparisons, *P* < 0.05 was considered significant. All statistical analyses were performed using STATA 14.

## Results

### Patient characteristics

Baseline demographic and clinical data of the entire cohort and their comparison between patients who did and did not develop pazopanib-induced HTN is illustrated in Table 3. The majority of patients had clear cell tumor histology (80%) and were started on the standard trial dose of pazopanib 800 mg daily (91%). Among the total cohort, more than half of patients had a prior nephrectomy (63%), current or past smoking history (60%), hypertension (60%), ECOG performance status score of 1 (54%), and renal insufficiency (57%). Gender, mean age at pazopanib initiation, and BMI levels were largely similar. A baseline ECOG performance status score of 0 was predominantly observed in the pazopanib-induced HTN group. Compared to patients who did not develop pazopanib-induced HTN, patients in the pazopanib-induced HTN group had a significantly higher mean baseline SBP (130.5 ± 10.9 mmHg vs. 121.7 ± 8.2; *P* = 0.01) and DBP (74.4 ± 8.5 mmHg vs. 69.7 ± 4.4; *P* = 0.045) along with a higher proportion of baseline ACEi or ARB usage (55% vs. 20%; *P* = 0.037) and higher incidence of pazopanib dose reduction during therapy (30% vs. 0%, *P* = 0.027). Other baseline CV disease and CV risk factors were not significantly different between the two groups. Median length of follow-up was 10 months (IQR: 3.1-19.4 months) for the total cohort and mortality occurred in 15/35 (43%) patients during the study period. Both characteristics were not significantly different between the two groups Table 2.

**Table 2:** Comparison of baseline characteristics in treatment-naïve metastatic renal cell carcinoma patients treated with pazopanib by occurrence of pazopanib-induced hypertension

Patient Characteristics	Total Cohort (*N* = 35)	Pazopanib-induced HTN (*N* = 20)	No Pazopanib-induced HTN (*N* = 15)	*P*-value
Male Gender	20 (57)	12 (60)	8 (53)	0.697
Age, years	61.9 ± 9.1	62.8 ± 10.4	60.7 ± 7.2	0.479
Pazopanib Therapy				
Initial dose 800 mg QD	32 (91)	18 (90)	14 (93)	1
Initial dose 400 mg QD	3 (9)	2 (10)	1 (7)	1
Dose reduction	6 (17)	6 (30)	0 (0)	0.027
Tumor Histology				
Clear cell	28 (80)	17 (85)	11 (73)	0.693
Papillary	4 (11)	2 (10)	2 (13)	0.712
Poorly differentiated	3 (9)	1 (5)	2 (13)	0.849
ECOG PS				
0	8 (23)	7 (35)	1 (7)	0.101
1	19 (54)	10 (50)	9 (60)	0.734
≥2	8 (23)	3 (15)	5 (33)	0.246
Nephrectomy	22 (63)	12 (60)	10 (67)	0.687
Heart Failure	2 (6)	1 (5)	1 (7)	1
LV Dysfunction	7 (20)	2 (10)	5 (33)	0.112
Diabetes Mellitus	15 (43)	9 (45)	6 (40)	0.775
Hypertension	21 (60)	14 (70)	7 (47)	0.173
Systolic BP, mm Hg	126.9 ± 10.8	130.5 ± 10.9	121.7 ± 8.2	0.010
Diastolic BP, mm Hg	72.3 ± 7.4	74.4 ± 8.5	69.7 ± 4.4	0.045
Dyslipidemia	17 (49)	10 (50)	7 (47)	0.851
GFR < 60 mL/min/1.73 m^2^	20 (57)	11 (55)	9 (60)	0.767
CAD/PAD	5 (14)	2 (10)	3 (20)	0.631
CVA/TIA	3 (9)	3 (15)	0 (0)	0.244
Thromboembolism	7 (20)	3 (15)	4 (27)	0.430
Smoker	21 (60)	14 (70)	7 (47)	0.297
BMI, kg/m^2^	29.4 ± 8.7	28.8 ± 6.8	30.2 ± 11.0	0.680
ACEIs/ARBs	14 (40)	11 (55)	3 (20)	0.046
Beta Blockers	11 (31)	5 (25)	6 (40)	0.467
Diuretics	7 (20)	5 (25)	2 (13)	0.672
CCBs	9 (26)	7 (35)	2 (13)	0.244
Statin	13 (37)	8 (40)	5 (33)	0.737
Deceased	15 (43)	9 (45)	6 (40)	0.767
Follow-up time, months	10.0 [3.1-19.4]	11.7 [4.2-20.9]	6.9 [2.1-17.7]	0.257

*HTN* hypertension, *QD* once daily, *ECOG* Eastern Cooperative Oncology Group performance status, *LV* left ventricular, *BP* blood pressure, *GFR* glomerular filtration rate, *CAD/PAD* coronary artery disease/peripheral arterial disease, *CVA/TIA* cerebrovascular accident/transient ischemic attack, *BMI* body mass index, *ACEI* angiotensin converting enzyme inhibitor, *ARB* angiotensin receptor blocker, *CCBs* calcium channel blocker. Data presented as a number with percent (%), mean ± standard deviation, or median [1st quartile-3rd quartile]

### Description of the Pazopanib-induced hypertensive response and associated risk factors

**Fig. 1:**
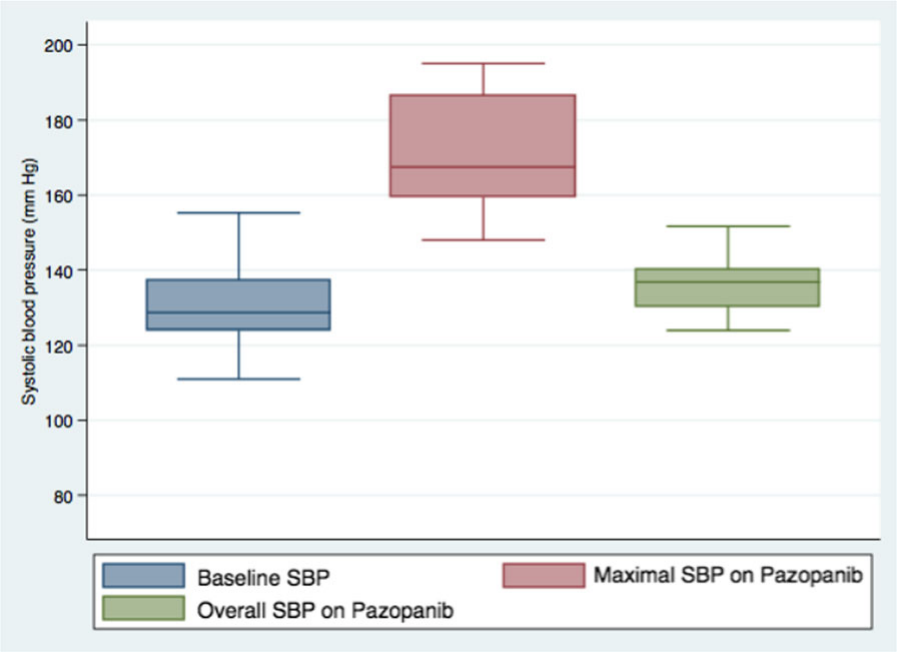
Median systolic blood pressure before and after pazopanib initiation in patients meeting criteria for pazopanib-induced hypertension (*N* = 20). Baseline median SBP is at the *far left* and is equal to 128.6 mmHg. Median maximal SBP is within the *middle box* and is equal to 167.5 mmHg. Median time to reach maximal SBP was 24.5 days as described in Table 3. Overall median SBP during pazopanib treatment is within the box on the far right and is equal to 136.8 mmHg. *Solid line* within each *box* represents the median. *Boxes* represent the interquartile range. *Bars* represent the range. SBP: systolic blood pressure

The majority of patients in our cohort (57%) developed pazopanib-induced HTN (Table 3). The overall median time from pazopanib start date to development of pazopanib-induced HTN was 24.5 days (IQR: 14.5-53.5 days). Of the 14 patients without preexisting HTN, 6 (43%) developed new-onset HTN with the median time to incident HTN of 19 days (range 7-53 days). Preexisting HTN was present in 21 patients, and 14 patients met criteria for pazopanib-induced HTN with a median time to event of 29.5 days (IQR 18-92 days). As Fig. 1 illustrates, there was a significant increase in SBP from baseline to the maximal measured during pazopanib treatment with an overall median SBP after pazopanib exposure 8.2 mmHg higher than baseline in pazopanib-induced HTN patients. A systolic blood pressure increase greater than 10 mmHg from baseline on at least two separate BP measurements during pazopanib therapy was seen in 25/35 (71%) patients in the entire cohort. Among patients in our cohort meeting criteria for pazopanib-induced hypertension, 15/20 (75%) patients had a systolic blood pressure increase greater than 10 mmHg from baseline on at least two separate BP measurements during pazopanib therapy. A total of 26 distinct episodes of either initiation or dose escalation of an antihypertensive occurred. ACEi/ARBs (46%) and CCBs (27%) accounted for the majority of these (Table 3).

**Table 3:** Features of pazopanib-induced hypertension in the 20 patients in which it developed

Parameter	Value	Range
Change in systolic BP (mm Hg)	8.2 [−3.7-18.4]	−54.6-26.3
Change in diastolic BP (mm Hg)	5.6 [0.4-11.4]	−8.3-18.2
Maximal systolic BP (mm Hg)	167.5 [159.5-186.5]	148-195
Maximal diastolic BP (mm Hg)	96 [92-106.5]	80-112
Time until pazopanib-induced HTN (days)	24.5 [14.5-53.5]	7-641
Antihypertensive dose increased or new agent added	17 (85)	
No preexisting HTN	6 (30)	
Class of antihypertensive started or intensified		
ACEIs or ARBs	12 (46)	
Beta-blockers	3 (12)	
Calcium channel blockers	7 (27)	
Diuretics	1 (4)	
Others*	3 (12)	

*BP* blood pressure, *HTN* hypertension, *n* number, *ACEI* angiotensin-converting enzyme inhibitor, *ARB* angiotensin receptor blocker

Data presented as a number with percent (%) or median [1st quartile-3rd quartile]

^*^Clonidine (*n* = 2) and hydralazine (*n* = 1)

Baseline SBP ≥ 130 mmHg (OR: 5.32; 95% CI: 0.94-29.99; *p* = 0.058) had a strong trend towards significance as a univariate predictor of development of HTN, and treatment with an ACEi or ARB (OR: 4.88; 95% CI: 1.05-22.84; *p* = 0.044) was a significant univariate predictor of development of pazopanib-induced HTN (Table 4). Neither characteristic maintained statistical significance on multivariate logistic regression. There was no significant association between development of pazopanib-induced HTN (*p* = 0.791) or ACEi/ARB treatment (*p* = 0.924) with overall survival.

**Table 4:** Univariate and Multivariable Logistical Regression Analysis of Risk Factors for Pazopanib-Induced Hypertension

	Univariate analysis		Multivariable analysis	
	OR	*P*-value	OR	*P*-value
Age ≥ 60 years	0.81	0.767		
Male (vs. female)	1.31	0.694		
Baseline SBP ≥ 130 mmHg	5.32	0.058	4.62	0.197
Antihypertensive therapy at baseline				
ACEIs or ARBs	4.88	0.044	4.31	0.075
Calcium channel blockers	3.5	0.160		
Diuretics	2.17	0.400		
Beta-blockers	0.5	0.348		
≥ 2 Antihypertensives	4	0.078		
Baseline CV Risk Factors				
Diabetes	1.23	0.767		
GFR < 60 ml/min/1.73m^2^	0.81	0.767		
BMI > 30 kg/m^2^	0.76	0.694		
Smoker^a^	2.12	0.281		
Oncologic Profile				
Prior Nephrectomy	0.75	0.687		
Pazopanib starting dose 800 mg	0.64	0.724		

*OR* odds ratio, *SBP* systolic blood pressure, *ACEI* angiotensin-converting enzyme inhibitor, *ARB* angiotensin receptor blocker, *CV* cardiovascular, *GFR* glomerular filtration rate, *BMI* body mass index, *ECOG* Eastern Cooperative Oncology Group performance status

^a^Current of past smoker

### Pazopanib-related cardiovascular Adverse events and associated risk factors

**Fig. 2:**
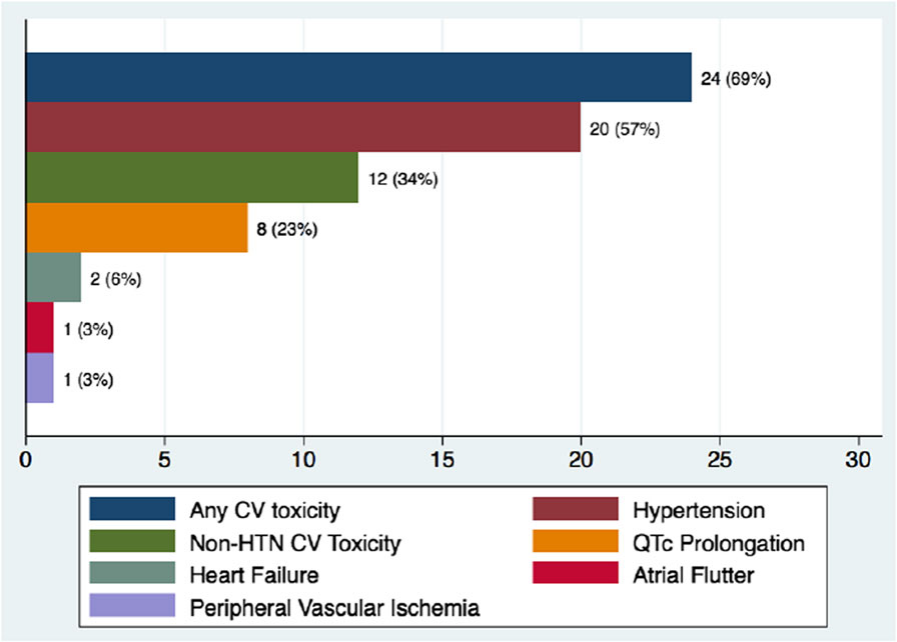
Incidence of cardiovascular toxicity by type in antineoplastic-naïve patients during pazopanib treatment. Twenty-four of 35 (69%) patients developed some form of CV toxicity with pazopanib treatment. After excluding HTN, 12/35 (34%) patients still developed a CV adverse event. Refer to Table 6 for clinical details of the 4 CV adverse events requiring hospitalization. CV: cardiovascular; HTN: hypertension; QTc: corrected QT interval

**Fig. 3:**
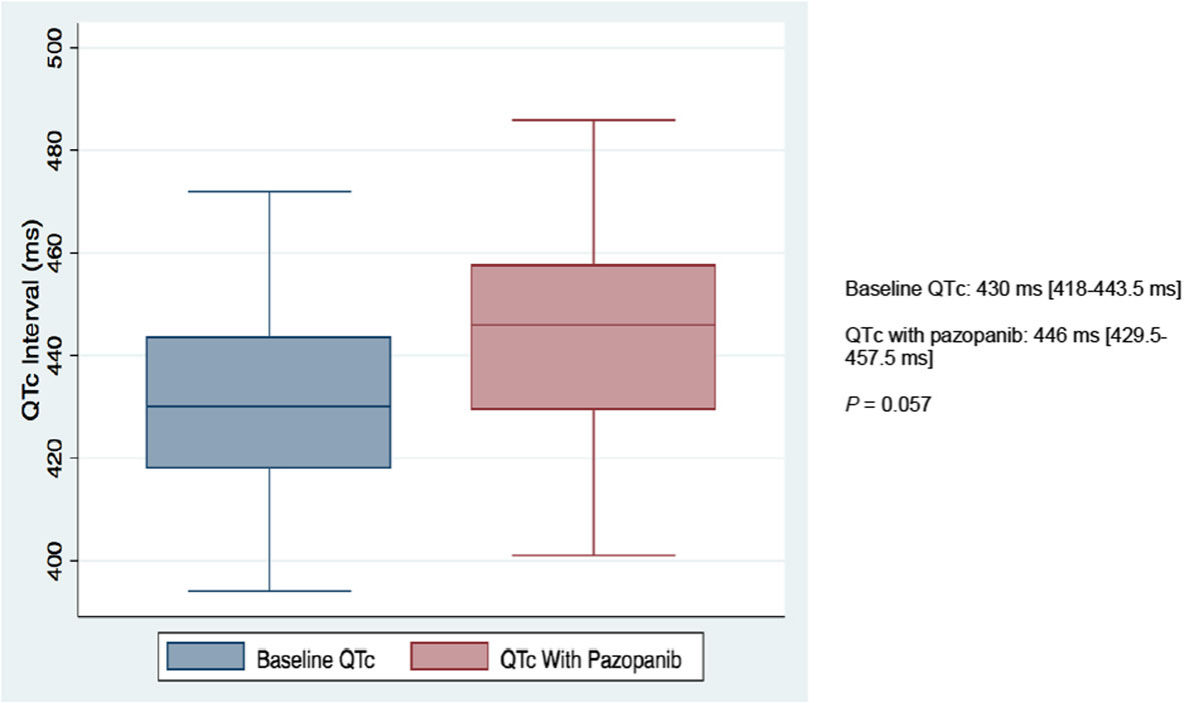
Comparison of median corrected QT intervals in 24 patients with electrocardiograms at baseline and after pazopanib initiation Line in each *box* represents the median while *boxes* represent the interquartile range. QTc values on the *right* represented as median [1st quartile-3rd quartile]. *P*-value obtained from match-paired Wilcoxon test (*N* = 24) assuming *P* < 0.05 represents significance. QTc: corrected QT interval; ms: milliseconds

**Fig. 4:**
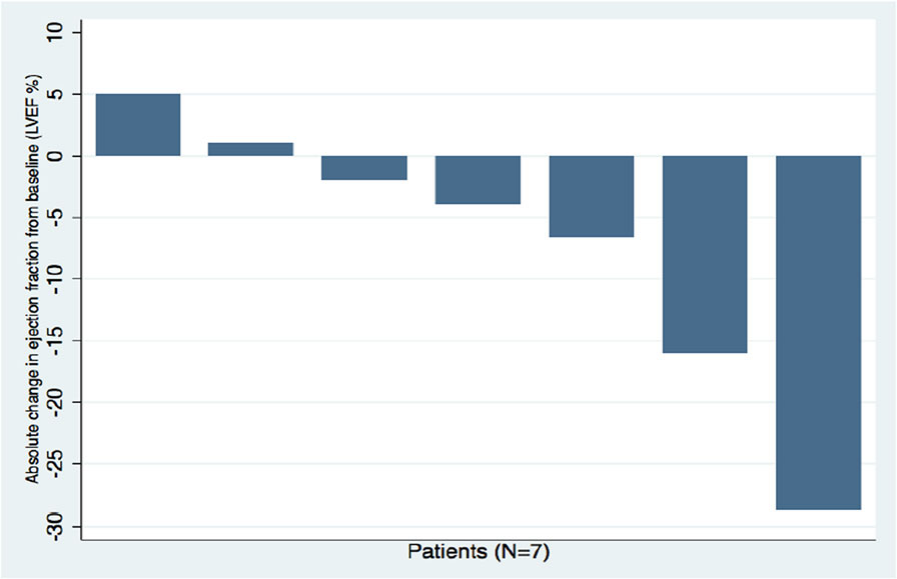
Absolute change in *left* ventricular ejection fraction from baseline in the seven patients with available echocardiograms as baseline and after pazopanib initiation. LVEF: left ventricular ejection fraction

Nearly 70% of patients in our study developed CV toxicity (Fig. 2, Table 5). When HTN was excluded, 12 of 35 patients (34%) still met criteria for developing a CVAE during the course of pazopanib treatment. QTc-interval prolongation was most common among these and represented 23% of all CVAEs. As shown in Fig. 3, pazopanib treatment was strongly associated with prolongation of the QTc-interval with a median increase of 16 ms (*p* = 0.057) detected in the 24 patients with baseline and treatment ECGs. Among the 7 total patients who had LVEF assessments before and after pazopanib exposure, an absolute decline in LVEF was observed in 5 patients (Figure 4). Among these 5 patients, two developed clinically significant declines in LVEF, defined as greater than 10%. This was not associated with concomitant uncontrolled hypertension at the time of diagnosis of LVEF decline with measured BPs of 114/79 and 140/75, both of which were below each respective patient’s baseline BP. The patient who developed the highest degree of LVEF decline (56 to 27% over a 2-month period after starting pazopanib) did have significant CV co-morbidities including LV dysfunction in form of grade II diastolic dysfunction determined on baseline echocardiography, Mobitz type 2 s-degree AV block for which he had a permanent pacemaker placed two years prior, diabetes mellitus, and a 55-year smoking history. High-grade CVAE requiring hospitalization and/or procedural intervention occurred in 4/12 (33%) patients with a non-HTN CVAE. Two patients developed acute HF within 30 days of pazopanib initiation and one progressed to fatal cardiogenic shock. Symptomatic atrial flutter requiring electrical cardioversion and ablation and leg ischemia requiring percutaneous revascularization were the two additional high-grade CVAEs (Table 6). With the exception of age and prior CVA/TIA, no significant differences were found between those who developed a CVAE and those who did not after excluding HTN (Table 7). Age ≥ 60 years was associated with non-HTN CVAE (OR: 8.72; 95% CI: 0.74-513.26; *p* = 0.105) though did not meet statistical significance as an independent predictor and on exploratory analysis, prior CVA/TIA was an additional risk factor (OR: 8.61; 95% CI: 0.86-infinite; *p* = 0.067) (Table 8). Statistical significance was also not maintained on multivariable adjusted logistic regression. There was no significant association between statin (*p* = 0.568) or beta-blocker (*p* = 0.714) therapy and survival.

**Table 5:** Description of overall cardiovascular toxicity observed with pazopanib treatment

Entire Study Population (n)	35
Any CV toxicity	24 (69%)
Any CV toxicity excluding hypertension	12 (34%)
Grade 1 QTc prolongation	6 (17%)
Grade 2 QTc prolongation	2 (6%)
Grade 3 heart failure	1 (3%)
Grade 5 heart failure	1 (3%)
Grade 3 atrial flutter	1 (3%)
Grade 3 peripheral ischemia	1 (3%)
Grade 2 hypertension	3 (9%)
Grade 3 hypertension	17 (49%)

*n* number, *CV* cardiovascular, *QTc* corrected QT interval. Refer to Table 1 for grading definitions. Results displayed as number of patients (% of all study patients)

**Table 6:** Clinical synopsis of all cardiovascular adverse events requiring hospitalization during pazopanib treatment

Age (years)	Gender	Pazopanib dose at time of event (mg)	Cardiac drugs at event	Time until event (days)	Type of Cardiovascular event	Past cardiac history and clinical synopsis	Notable cardiac diagnostic findings	Outcome
67	Male	800	None	30	Cardiogenic Shock	H/O grade II diastolic dysfunction and 2nd degree AV block SP PPM. SB at rest and LE edema. BNP = 2567. Troponin normal.	LVEF decline noted from 56% pre-pazopanib to 27% after pazopanib. No wall motion abnormalities detected on TTE.	Pazopanib discontinued on admission. Treated with furosemide with initial improvement but developed cardiogenic shock and subsequent PEA arrest and death.
60	Female	800	None	16	Acute HFrEF	No previous cardiac history. SB, orthopnea. JVP elevated. BNP = 3712. Troponin normal.	LVEF = 10% on CMR. No previous LVEF available for comparison. Mid-myocardial fibrosis and elevated extracellular volume fraction of 35% (normal <29%) suggestive of non-ischemic cardiomyopathy	Pazopanib discontinued. Treated with IV furosemide and started GDMT. Re-admitted two weeks later for hypotension and uncontrolled cancer-related pain. Due to hypotension was unable to tolerate GDMT for HFrEF.
59	Male	800	Atorvastatin, furosemide, ramipril, pioglitazone, metformin, warfarin	37	Atrial flutter	H/O HTN, DM, HLD developed new-onset atrial flutter 12 days after spinal and hip surgery for metastatic bone cancer.	LVEF 55-60% on TEE. EPS confirmed the mechanism of tachycardia to be right atrial flutter within the cavo-tricuspid isthmus.	Successful TEE-guided DCCV restored normal sinus rhythm, followed by ablation. Patient deceased nine months after event due to progression of malignancy.
85	Female	800	Aspirin, diltiazem, simvastatin	662	Ischemic Left Lower Extremity	H/O CAD, CVA, HLD, HTN, PAD with two prior percutaneous interventions to lower extremities preceding pazopanib initiation. Developed left leg pain. Non-emergent presentation.	Totally occluded left popliteal artery. Multiple 70-80% stenotic lesions of the left superficial femoral artery. Occlusion of the left peroneal artery.	Successful percutaneous intervention. Pazopanib was continued without any recurrent ischemic events for the remainder of the study period.

*H/O* history of, *SP* status post, *AV* atrioventricular, *PPM* permanent pacemaker, *SB* shortness of breath, *LE* lower extremity, *BNP* brain natriuretic peptide, *LVEF* left ventricular ejection fraction, *TTE* transthoracic echocardiogram, *PEA* pulseless electrical activity, *HFrEF* heart failure with reduced ejection fraction, *JVP* jugular venous pressure, *CMR* cardiac magnetic resonance, *IV* intravenous, *GDMT* guideline directed medical therapy, *HTN* hypertension, *DM* diabetes mellitus, *HLD* hyperlipidemia, *TEE* transesophageal echocardiogram, *EPS* electrophysiology study, *DCCV* direct-current cardioversion, *CAD* coronary artery disease, *CVA* cerebrovascular disease, *PAD* peripheral arterial disease

**Table 7:** Comparison between patients who developed pazopanib-related cardiovascular toxicity after excluding hypertension

Patient Characteristics	Pazopanib-induced non-HTN CV toxicity (*N* = 12)	No Pazopanib-induced non-HTN CV toxicity (*N* = 23)	*P* Value
Male Gender	5 (42)	15 (65)	0.282
Age, years	66 [61-71]	57 [52-65]	0.006
LVEF, %	^*^60 [59-67]	^**^62.5 [59-66]	0.712
Systolic BP, mm Hg	124.1 [120.8-130.8]	127.7 [122-132.6]	0.728
Diastolic BP, mm Hg	70.0 [65.9-73.5]	72 [67.9-79.3]	0.297
Heart Failure	0 (0)	2 (9)	0.536
LV Dysfunction	4 (33)	3 (13)	0.200
Diabetes Mellitus	5 (42)	10 (43)	1
Hypertension	7 (58)	14 (61)	1
Dyslipidemia	8 (67)	9 (39)	0.164
GFR < 60 mL/min/1.73 m^2^	9 (75)	11 (48)	0.163
CAD/PAD	3 (25)	2 (9)	0.313
CVA/TIA	3 (25)	0 (0)	0.034
Dysrhythmia	3 (25)	4 (17)	0.670
Thromboembolism	1 (8)	6 (26)	0.380
Smoker*, n (%)	7 (58)	13 (57)	1
BMI (kg/m^2^)	27.6 [23.6-31.8]	28.5 [21.9-32.9]	0.627
ACEIs or ARBs	6 (50)	8 (35)	0.383
Beta Blockers	4 (33)	8 (35)	1
Statin	6 (50)	7 (30)	0.256
Pazopanib dose reduction	3 (25)	3 (13)	0.391
Follow-up time, months	11.7 [4.2-20.9]	6.9 [2.1-17.7]	0.509

*ACEI* angiotensin converting enzyme, *ARB* angiotensin receptor blocker, *BP* blood pressure, *BMI* body mass index, *CAD/PAD* coronary artery disease/peripheral arterial disease, *CCBs* calcium channel blockers, *ECOG* Eastern Cooperative Oncology Group performance status, *LVEF* left ventricular ejection fraction, *GRF* glomerular filtration rate; Data presented as a percent (%) or median [1st quartile-3rd quartile]

^*^ *N* = 9; ^**^ *N* = 16

^a^Current or prior smoking history

**Table 8:** Univariate and Multivariate Variables Associated with Pazopanib-Related Non-Hypertension Cardiovascular Toxicity

	Unadjusted (univariate analysis)		Adjusted (multivariate analysis)^a^	
	OR	*P*-value	OR	*P*-value
Age ≥ 60 years	15.79	0.006	8.72	0.105
Male (vs. female)	0.39	0.329		
Hypertension	0.90	1		
Diabetes	0.93	1		
Dyslipidemia	3.01	0.233		
CVA/TIA^b^	8.61	0.067	2.77	0.430
CAD/PAD	3.36	0.418		
GFR < 60 ml/min/1.73m^2^	3.16	0.236		
LV Dysfunction	3.21	0.327		
Dysrhythmia	1.56	0.906		
BMI > 30 kg/m^2^	0.93	1		
Smoker	1.07	1		
ACEi/ARB use	1.84	0.608		
Beta Blocker use	1.14	1		
Statin use	2.23	0.441		

*OR* odds ratio, *CI* confidence interval, *CVA/TIA* cerebrovascular accident/transient ischemic attack, *CAD/PAD* coronary artery/ peripheral artery disease, *GFR* glomerular filtration rate, *LV* left ventricular, *BMI* body mass index, *ACEI* angiotensin converting enzyme, *ARB* angiotensin receptor blocker

^a^Model includes gender, preexisting CVA/TIA, dyslipidemia, and GFR < 60 ml/min/1.73m^2^

^b^CVA/TIA was a perfect predictor for a non-hypertension cardiovascular event. *P* values based on exact logistic regression

## Discussion

The major findings in this study of antineoplastic-naïve mRCC patients newly started on pazopanib includes: (1) a strikingly high proportion (69%) of patients developing a form of CV toxicity ranging from asymptomatic cardiac repolarization abnormalities on ECG to fatal cardiogenic shock; (2) a marked and rapid hypertensive response corresponding to a higher observed rate of high-grade HTN in our cohort than previously reported with pazopanib; and (3) an absolute decline in LVEF after pazopanib exposure in 5/7 (71%) patients who had LVEF assessments before and after treatment.

### Pazopanib-induced hypertension

Any grade of pazopanib-induced HTN was seen in 20/35 (57%) patients, exceeding the reported incidence rates of 36-46% [4, 19, 20]. A marked hypertensive response (>20 mmHg increase in SBP or DBP) was observed in these 20 patients. Notably 85% of the patients in our study met CTCAE v4.0 criteria for grade 3 HTN, which is a significantly higher proportion than the 4-7% incidence reported in earlier phase II/III clinical trials [4, 20]. Two contributing factors may explain this discrepancy. First, early pazopanib trials from which much of the data comes from excluded patients with comorbidities such as poorly controlled HTN or underlying CV disease that may portend them to a more drastic BP response. Secondly, those earlier clinical trials assigned HTN grades based on CTCAEv3.0 definitions that were not aligned with the standard definition of HTN established by JNC guidelines. If using CTCAE v3.0 where HTN was defined as BP greater than 150/100, 15/35 (42%) of patients in our cohort would meet criteria for developing pazopanib-induced hypertension. This is more consistent with previously reported data on the incidence of pazopanib-induced HTN and suggests that early trials on pazopanib may have under-reported the incidence of HTN with its use. Development of pazopanib-induced HTN was rapid with more than half of cases occurring within 25 days of pazopanib initiation. This is consistent with prior studies on HTN related to VSPIs [30, 31]. The true time to development of pazopanib-induced HTN may actually be shorter than what we observed given findings from a prospective study with sorafenib where ambulatory BP surveillance demonstrated BP elevation during the first 24 h of treatment [30]. After peak BP levels were achieved, we saw a subsequent decline towards baseline. This likely represents the effect of more intensive antihypertensive therapy after treating clinicians recognized pazopanib-induced HTN. A similar pattern has been observed in prior studies involving multiple agents within the VSP inhibitor class [30–32]. Not surprisingly, preexisting hypertension has been found to be a risk factor for VSPI-induced HTN [19, 31, 33]. Our data are consistent with these previous findings, with an association existing between the development of pazopanib-induced HTN and presence of a baseline prehypertension. Of note, 17/20 (85%) patients with preexisting HTN had adequate baseline BP control (<140/90 mmHg) using JNC-8 guidelines and BP targets before initiation of VSPIs proposed in prior studies [28, 31]. Despite the vast majority of preexisting HTN patients in our study achieving these targets, they still developed pazopanib-induced HTN with strikingly high magnitudes of BP elevation. Given that mRCC patients have relatively limited life expectancies, acute complications from uncontrolled HTN have historically been of particular concern with VSPI initiation [34, 35]. However, advances in the treatment of mRCC have improved survivorship to the point where median overall survival in pazopanib-treated patients is now 22.9 months and improves to 42.5 months in patients with favorable oncologic features [4, 36]. In our study, all 6 of the patients requiring pazopanib dose reduction also developed pazopanib-induced HTN. A lower BP target prior to initiating pazopanib may attenuate its drastic hypertensive effects and optimize oncologic care in addition to reducing the risk of longer-term complications of HTN that may become more apparent as further advancements in the development of targeted antineoplastic agents are made.

Two recent studies have shown improved overall survival rates in mRCC patients on VSPI therapy undergoing concomitant treatment with an ACEi or ARB [37, 38]. Our data did not demonstrate a similar ACEi/ARB survival benefit. This may be due to our cohort being limited to only pazopanib-treated mRCC patients given that exploratory analysis from a large secondary pooled analysis of two RCTs also did not find a survival advantage in pazopanib-treated patients on ACEi/ARB therapy [39]. It has been proposed that ACEi/ARBs act synergistically with VSPIs to enhance their antineoplastic effect [38]. It is unclear from a mechanistic standpoint why the same degree of potentiation with pazopanib is not seen and also highlights the need to study VSPI agents individually to better characterize their clinical effects.

### Pazopanib-related cardiac toxicity

After excluding HTN, 12/35 patients (31%) still met criteria for developing a CVAE which is significantly higher than what was described in clinical trials but is consistent with a similar CV-focused study in a clinical setting where 13/43 (30%) pazopanib-treated patients developed a non-HTN CVAE [40]. A few differences in each study, however, are worth noting. By design, our cohort of mRCC patients was treatment naïve with no prior exposure to potentially cardiotoxic antineoplastic agents. This was by design given the uncertainty of potential long-term cardiotoxic effects with some of the novel agents used in the treatment of mRCC. Secondly, because the other investigators had an established CV monitoring protocol for TKI-treated patients, they were able to utilize cardiac biomarkers as a measure of cardiotoxicity and had a higher proportion of patients with LVEF assessments at baseline and during treatment that led to a higher detection rate of low grade HF as defined in Table 1. Conversely, our study included cardiac conduction abnormalities and peripheral ischemia as CVAEs while theirs did not. The overall severity of pazopanib-related CVAEs that we observed appears to be higher by comparison. In particular, we observed two HF events and both were high-grade, with one resulting in death and the other in pazopanib discontinuation (Tables 5, 6) compared to all HF events being grade 1 or 2 severity in this earlier study. The development of high-grade HF in 2/35 (6%) patients in our study is almost six times the rate reported in early clinical trials [20]. Though this may be reflective of the difference in number of study participants, it is also possible the higher rate we observed is related to our cohort consisting of patients outside the clinical trial setting, with a higher burden of comorbidities.

Our data suggests that age greater than 60 years may increase the risk for a pazopanib-related non-HTN CVAE. This is not surprising given CV risk increases with age even in healthy adults given the higher prevalence of comorbid conditions such as HTN, DM, and atherosclerotic disease. There may be a potential mechanistic link to this observation given the experimental finding that older mice treated with the TKI imatinib experience more severe cardiotoxicity as a result of age-dependent increase in oxidative stress [41]. It is also worth noting that the most well-recognized cardio-oncology clinical guidelines specifically recommend increased attention to cardiac function surveillance for patients ≥60 years old treated with anthracyclines and/or trastuzumab given limited data on this population [42]. Given the uncertainty of the full scale of cardiotoxicity with pazopanib and other novel VSPIs, prospective clinical studies assessing the benefit of standardized CV functional assessment in this patient population is warranted.

QTc interval prolongation with small molecule TKIs such as pazopanib has been postulated to be related to “off target” blockade of the HERG K+ channel and has the potential to increase the risk of potentially life-threatening unstable ventricular dysrhythmias [43, 44]. We observed a higher proportion of patients developing QTc intervals >500 ms than what was reported in clinical trials (6% vs. <2%). Given that cancer patients are prone to diarrhea- and vomiting-related electrolyte derangements, these may have been identified and corrected at higher rates in the clinical trial setting where study participants are monitored with more frequent lab testing at regular intervals. Another contributing factor may have been a higher degree of concomitant use of QTc-prolonging medications such as antiemetics and psychotropics in our cohort. Of the 9 patients in our study that developed CV toxicity in the form of QTc prolongation, none developed Torsades de Pointes or another life-threatening ventricular dysrhythmia. Though these events are rare (<1% incidence in clinical trials), the significant morbidity and mortality rate they pose warrants regular ECG monitoring during pazopanib treatment.

### Limitations

This was a retrospective, observational single center study with information obtained from the EMR. As with all analyses using EMRs, potential introduction of unidentifiable sources of bias warrants consideration. Preexisting HTN may have been affected by gender, age, patient comorbidities, and concurrent medication use. We attempted to address this problem by using multivariate risk adjustment, but unmeasured variables inherently cannot be accounted for in this study design. Variability in hospital coding practices and physician documentation may have resulted in underestimation of some comorbidities. The use of a standardized EMR data extraction template and physician review of medical records was employed to minimize this factor.

The lack of standardization of echocardiographic monitoring of LV function was evident in our study cohort, with a large proportion of patients not having regular surveillance of LV function during pazopanib treatment and 25/35 (71%) patients having a baseline LVEF assessment. This makes it difficult to draw significant conclusions about the echocardiographic data collected though a trend toward LVEF reduction with pazopanib treatment was observed. Variability in physician echocardiograph interpretation was also a source of potential bias. We attempted to minimize this by collecting strictly quantitative data from echocardiography reports. The lack of echocardiographic screening prior to pazopanib initiation in nearly 30% of our total cohort may be reflective of under-recognition of the CV risk pazopanib poses. Considering the marked hypertensive response with pazopanib therapy, implementation of a standardized CV risk assessment protocol that incudes echocardiographic screening for these patients is warranted. The lack of standardized measurement of cardiac biomarkers in our patient cohort is also a limitation as it may have caused under-detection of pazopanib-induced subclinical CV toxicity. Incorporating cardiac biomarker measurement into CV risk assessment and surveillance protocols in clinical practice before and during pazopanib therapy should thus be considered.

The size of the patient cohort that was utilized may limit our generalizability. A larger total cohort may have allowed for identification of more predictors of CVAEs and perform more robust survival analysis. By study design, we excluded patients treated with any other systemic antineoplastic agent including TKIs such as sunitinib, sorafenib, cabozantinib or axitinib. Since our focus was to assess CV risk factors and cardiotoxicity associated strictly with pazopanib in the hopes of possibly elucidating mechanistic links specific to pazopanib, this would have introduced a major confounder into our study. However, given the known overlap in receptor affinities among the VSPIs, our findings could potentially be applied to other agents in this class. Lastly, the study population was composed of patients from a single healthcare system. As a result, the level of generalizability is not entirely clear. However, the OSUWMC is a large tertiary care referral center and the population of patients encountered likely resembles most large medical centers.

## Conclusions

This is the first study that exclusively examined pazopanib-induced CV effects in antineoplastic-naïve mRCC patients in a clinical setting. Our findings suggest that pazopanib possesses a multifaceted cardiovascular toxicity profile which includes cardiomyopathy ranging from asymptomatic reduction in LVEF to fatal cardiogenic shock, cardiac repolarization disturbances manifested by QTc-interval prolongation, and a striking hypertensive response predominantly within 30 days of starting pazopanib that was associated with pazopanib dose reduction.

Preexisting CV disease has been identified in as many as 35% of renal cell cancer patients in the US [45]. Combined with the fact the cancer survivorship continues to improve with the rapid evolution of targeted therapies such as pazopanib, the intersection between cardiovascular and oncologic disease will likely continue to expand. Standardization of CV risk stratification prior and cardiac surveillance in patients undergoing treatment with pazopanib and other VSPIs can optimize oncologic care while minimizing potentially avoidable CV risk. The findings presented here are hypothesis generating and need to be validated in larger, prospective, cardiovascular-focused studies.

Future studies can be focused on early detection and preventive management of subclinical CV disease associated with pazopanib and other agents within the VSPI class. This may include assessing the utility of more sensitive cardiac diagnostic modalities such as strain imaging for detection of subclinical LV dysfunction with novel VSPIs as has been shown with trastuzumab, anthracyclines, and taxanes [46]. Investigation of whether concurrent BB and/or ACEi/ARB use during pazopanib treatment imparts a cardioprotective effect as has been demonstrated in anthracycline-induced cardiomyopathy is another area that may warrant further investigation [47, 48].

## Abbreviations

ACEI: angiotensin converting enzyme inhibitor
AEs: adverse effects
ARB: angiotensin receptor blocker
BMI: body mass index
BP: blood pressure
CAD/PAD: coronary artery disease/peripheral arterial disease
CCBs: calcium channel blocker
CI: Confidence interval
CTCAE v4.0: National Cancer Institute’s Common Terminology Criteria for Adverse Events version 4.0
CV: Cardiovascular
CVA/TIA: cerebrovascular accident/transient ischemic attack
CVAE: Cardiovascular adverse event
DBP: Diastolic blood pressure
ECOG: Eastern Cooperative Oncology Group performance status
EMR: Electronic medical record
GFR: glomerular filtration rate
HF: Heart failure
HTN: hypertension
ICD-9/10: International Classification of Diseases – 9 and 10
IQR: Interquartile range
JNC-8: Joint National Committee on Prevention, Detection, and Treatment of High Blood Pressure, 8th edition
LV: Left ventricle
LVEF: Left ventricular ejection fraction
mRCC: Metastatic renal cell carcinoma
OR: Odds ratio
OSUWMC: Ohio State University Wexner Medical Center
PDGFR: Platelet-derived growth factor receptor
QTc: Corrected QT-interval
SBP: Systolic blood pressure
TKI: Tyrosine kinase inhibitor
VEGF: Vascular endothelial growth factor
VSPI: VEGF signaling pathway inhibitor
